# Supplementary material for: Evaluating the diagnostic utility of the slide agglutination method for brucellosis in Saudi Arabia: A retrospective study at International Medical Center, Jeddah, Saudi Arabia
Source: PLoS Negl Trop Dis. 2025 Oct 6;19(10):e0013206. doi: 10.1371/journal.pntd.0013206 (PMC12500134; doi:10.1371/journal.pntd.0013206)
Supplement: Attachment — Submitted filename: Response sheet.docx [file pntd.0013206.s005.docx]

**Comment 1.** We do not publish any copyright or trademark symbols that usually accompany proprietary names, eg ©,  ®, or TM  (e.g. next to drug or reagent names). Therefore please remove all instances of trademark/copyright symbols throughout the text, including:
- ® on pages: Micropath® on line number 138, and 238.

**Response:** All instances of trademark/copyright symbols have been removed from the manuscript.

**Comment 2.** Please ensure that all Figure files have corresponding citations and legends within the manuscript. Currently, " Figure 4 and 5 " in your submission file inventory does not have an in-text citation. If the figure is no longer to be included as part of the submission, please remove it from the file inventory.

**Response:** Figure 4 and 5 have been removed from the file inventory.

**Comment 3.** We have noticed that you have uploaded Supporting Information files, but you have not included a list of legends. Please add a full list of legends for your Supporting Information files after the references list.

**Response:** Legends for supporting information have been added.

**Comment 4.** Please include a completed 'Competing Interests' statement in the text box when submitting your production task, including any COIs declared by your co-authors, written in full sentences.
If you have no competing interests to declare, please state "The authors have declared that no competing interests exist". You may also provide an updated statement via email.

**Response:** We confirm that none of the authors have any competing interests to declare. Accordingly, we have included the following statement in the text box during the production task submission and have also added it to the manuscript:

"The authors have declared that no competing interests exist."
