# Supplementary material for: Evaluating the diagnostic utility of the slide agglutination method for brucellosis in Saudi Arabia: A retrospective study at International Medical Center, Jeddah, Saudi Arabia
Source: PLoS Negl Trop Dis. 2025 Oct 6;19(10):e0013206. doi: 10.1371/journal.pntd.0013206 (PMC12500134; doi:10.1371/journal.pntd.0013206)
Supplement: Attachment — Submitted filename: Response_sheet_auresp_2.docx [file pntd.0013206.s006.docx]

**22^nd^ April 2025**

**Methods**

**Reviewer #1:**

**Comment 1:** This was a retrospective observational study to evaluate the diagnostic performance of the Slide Agglutination Method (SAM). The investigators wanted to access the sensitivity, specificity, and overall performance of SAM compared to the gold standard blood cultures and Brucella serology. On that score the objective is well stated and clear. The design is very simple, all records of people aged 18 years and older who reported to the health facility from 2019 to 2022 were analyzed. Sample size was not calculated and mentioned so there is no way to tell. There is evidence of ethical approval.

**Response:** Sample size calculation has been provided (Page 5-6, Line 112-117).

**Comment 2:** Based on the objectives, the statistical analysis done is adequate.

**Response:** Thank you for your positive feedback.

**Comment 3:** It is stated that A Standard Wright test (SWT) ≥ 160 or a tube agglutination test with antihuman globulin (AHG TAT) ≥ 160 was used as the reference standard. Can they clarify this? Was this done for this study with archived blood samples or this was also part for the data retrieved for analysis in this study.

**Response:** Thank you for the opportunity to clarify. In this retrospective review, we did not re-run agglutination assays on stored sera. Instead, all tests were those originally performed by the microbiology laboratory at the time of patient evaluation and were directly retrieved from the laboratory information system. This has been noted in the manuscript (Page 5, Line 101-103; Page 5, Line 110-111).

**Reviewer #2:**

**Comment 1:** Question 1: Not clear blood culture specific operation details, such as acquisition, training time is extended to 14 days in order to improve the sensitivity or higher).

**Response:** Detailed methodology for blood culture has been provided (Page 6, Line 120-129).

**Comment 2:** Question 2: SAM's interpretation standards (such as "agglutination 1:80 or more positive") are consistent with the reagent instruction? Additional quotes or explanations are required. Suggestion: replenish blood culture and SAM's standardized operation process, ensure the method repeatability.

**Response:** The agglutination threshold of ≥1:80 is based on previously published literature, and the relevant references have now been added. Additional details about the blood culture procedures and the standardized SAM protocol have also been included to clarify consistency and ensure methodological repeatability (Page 6, Line 120-129; Page 7, Line 138-148).

**Reviewer #3:**

**Comment 1:** The objectives are clear but the hypothesis is not specifically formulated.

**Response:** This suggestion has been incorporated (Page 4-5, Line 91-93).

**Comment 2:** The authors do not specify the antibody titers for serology tests (IgG or IgM) that are considered positive in the Materials and Methods section. They also do not clarify whether a positive result for IgM or IgG alone is considered positive or whether positivity for both is necessary for the diagnosis.

**Response:** This information has been incorporated (Page 6, Line 133-135).

**Comment 3:** The authors also state that serology offers high sensitivity and specificity, but they do not quantify this sensitivity or specificity or provide any citations to support their claim (line 112). It should be noted that other publications have described false positives in non-endemic regions.

**Response:** We have now quantified the sensitivity and specificity of the Brucella IgG/IgM ELISA and added the supporting citation in the Methods section (Page 6, Line 135-136).

**Comment 4:** Regarding the patient sample, were some patients with chronic brucellosis? The authors should clarify and justify this point. The accuracy of serology and cultures may vary in cases of chronic brucellosis. On the other hand, it would be strange if in a retrospective sample based on serology tests there were no cases of chronic brucellosis.

**Response:** We have clarified that our cohort includes both acute and chronic brucellosis cases, defined chronicity as ≥1 year of symptoms. We also noted that culture yields and serology titres are lower in chronic presentations (Page 5, Line 104-109).

**Comment 5:** No sample size calculation is performed.

**Response:** Sample size calculation has been provided (Page 5-6, Line 112-117).

**Comment 6:** The authors should clarify when a IgG or IgM serology was considered positive.

**Response:** This information has been incorporated (Page 6, Line 133-134).

**Results**

**Reviewer #1:**

**Comment 7:** The results are well presented and the tables are clear

**Response:** Thank you for the positive feedback.

**Reviewer #2:**

**Comment 1:** Question 3: In table 4 "True positive (%)" column value conflict (such as blood culture of "True positive = 24.3%" and "Sensitivity = 40.9%" logic). Need to recalibrate calculations or amend tables.

**Response:** Table 4 (now Table 3) has been revised.

**Comment 2:** Question 4: Did not talk about SAM's performance in chronic cases (only mention "should be combined with other detection" but did not provide data support).
Suggestion: correct form, complement calculation formula in detail. If chronic cases were included in the study, the sensitivity of SAM should be analyzed separately. If not, it should be clearly stated in the limitation.

**Response:** As correctly noted, chronic brucellosis cases were not distinguished in our dataset. This limitation has been explicitly acknowledged in the manuscript:

“Chronic brucellosis cases were not distinguished in the dataset; therefore, the sensitivity of the Slide Agglutination Method (SAM) in chronic presentations could not be analyzed separately. This limits the study’s ability to determine SAM’s diagnostic performance across the full clinical spectrum of brucellosis” (Page 14, Line 283-286).

**Reviewer #3:**

**Comment 1:** The results section begins by describing the turnaround time of the different techniques. It is probably better to begin with a description of the patient sample, their symptoms, risk factors, and treatment. (line 167 et seq.).

**Response:** This suggestion has been incorporated (Page 8-9, Line 175-188)

**Comment 2:** Why is the outcome unknown for 56.8% of cases?

**Response:** We agree that the high proportion of unknown outcomes requires clarification. Because our study was based on retrospective chart review, follow-up data were missing or incomplete for 21 patients (56.8%). We have added this explanation in the manuscript (Page 9, Line 181-182)**.**

**Comment 3:** Table 3 is irrelevant. What is already stated in the text is sufficient.

**Response:** Table 3 has been removed.

**Comment 4:** Figure 1 contributes nothing to the article's content and can be deleted.

**Response:** Figure 1 has been removed.

**Comment 5:** Figure 3 is irrelevant and can be deleted.

**Response:** Figure 3 has been removed.

**Conclusions**

**Reviewer #1:**

**Comment 1:** Based on the objectives, the conclusions are justified. The limitations of the analysis are not clearly articulated however, the study does not provide any new insights other than what is already known or what is expected hence the public health relevance is not clear. The authors could provide some context of the prevalence of brucellosis in the study region to make a case for the public health relevance.

**Response:** Conclusion has been modified as per the suggestion (Page 15, Line 291-300).

**Reviewer #2:**

**Comment 1:** Not compared with similar studies (such as 10-12 only general references mentioned "sensitivity", but not concrete analysis the reasons for differences).

**Response:** This suggestion has been incorporated (Page 12-13, Line 232-243).

**Comment 2:** low sensitivity of blood cultures (40.9%), insufficient explanation (such as whether because of sampling time, antibiotic use, or culture differences?).
Suggestion: Increase compared with recent research (e.g., Meta data analysis or area), heterogeneity of SAM performance are discussed. The reasons for the low sensitivity of blood culture (such as disease stage and operational factors) were analyzed in combination with the literature.

**Response:** This suggestion has been incorporated (Page 13, Line 240-243; Page 13-14, Line 257-264).

**Reviewer #3:**

**Comment 1:** In the conclusion, the authors state that "…confirmatory testing remains essential for comprehensive diagnosis, especially in chronic cases" (lines 250-251). This statement is not supported by the data provided by the authors in this study.

**Response:** The original statement was intended to highlight a general diagnostic consideration and acknowledge the limitations of SAM in chronic brucellosis, rather than to draw a conclusion directly from our dataset. To avoid potential misinterpretation, we have revised this sentence in the conclusion to improve clarity (Page 15, Line 295-297).

**Comment 2:** In the discussion section (lines 207-210), it is stated that "Our results showed that the SAM has an average turnaround time of 2.32 hours for Brucella-positive cases, which is significantly faster than blood culture..." This sentence is inappropriate since the authors do not compare techniques in the results but compare positive and negative results for the same technique.

**Response:** This line has been removed from the manuscript.

**Comment 3:** The results could be helpful in endemic areas, but nothing is mentioned regarding non-endemic areas

**Response:** As suggested, we have added a statement in the discussion acknowledging the potential limitations of applying SAM in non-endemic regions (Page 14, Line 277-281).

**Comment 4:** Do the authors discuss how these data can be helpful to advance our understanding of the topic under study? The authors should discuss this point better and refer to the results of more studies on the outcome of serologies and other laboratory methods.

**Response:** This suggestion has been incorporated (Page 13, Line 246-251).

**Editorial and Data Presentation Modifications?**

**Reviewer #1:**

**Comment 1:** Line 171: B. abortus and B. melitensis (should be in italics), same with figure 4
Table 2: The frequency and percentage can be in the same column eg 36 (97.3%)

**Response:** This suggestion has been incorporated.

**Summary and General Comments**

**Reviewer #1:**

**Comment 1:** The paper is very well written and the objectives are clearly stated. The novelty and significance has not been well articulated. What new information is this study adding to the already existing knowledge on the subject matter and how is this going to change policy in Saudi Arabia?

**Response:** This suggestion has been incorporated (Page 14, Line 271-276).
